# Biosurveillance in Central Asia: Successes and Challenges of Tick-Borne Disease Research in Kazakhstan and Kyrgyzstan

**DOI:** 10.3389/fpubh.2016.00004

**Published:** 2016-02-01

**Authors:** John Hay, Kenneth B. Yeh, Debanjana Dasgupta, Zhanna Shapieva, Gulnara Omasheva, Pavel Deryabin, Talgat Nurmakhanov, Timur Ayazbayev, Alexei Andryushchenko, Asankadyr Zhunushov, Roger Hewson, Christina M. Farris, Allen L. Richards

**Affiliations:** ^1^State University of New York, Buffalo, NY, USA; ^2^MRIGlobal, Rockville, MD, USA; ^3^Scientific Practical Center for Epidemiological Expertise and Monitoring, Almaty, Kazakhstan; ^4^Kazakh Scientific Center for Quarantine and Zoonotic Diseases, Almaty, Kazakhstan; ^5^Uralsk Anti-Plague Station, Uralsk, Kazakhstan; ^6^Biotechnology Institute, Bishkek, Kyrgyzstan; ^7^Public Health England, Salisbury, UK; ^8^Naval Medical Research Center, Silver Spring, MD, USA

**Keywords:** tick-borne diseases, TBE virus, *Rickettsia*, *Coxiella*, Kazakhstan, Kyrgyzstan, biosurveillance

## Abstract

Central Asia is a vast geographic region that includes five former Soviet Union republics: Kazakhstan, Kyrgyzstan, Tajikistan, Turkmenistan, and Uzbekistan. The region has a unique infectious disease burden, and a history that includes Silk Road trade routes and networks that were part of the anti-plague and biowarfare programs in the former Soviet Union. Post-Soviet Union biosurveillance research in this unique area of the world has met with several challenges, including lack of funding and resources to independently conduct hypothesis driven, peer-review quality research. Strides have been made, however, to increase scientific engagement and capability. Kazakhstan and Kyrgyzstan are examples of countries where biosurveillance research has been successfully conducted, particularly with respect to especially dangerous pathogens. In this review, we describe in detail the successes, challenges, and opportunities of conducting biosurveillance in Central Asia as exemplified by our recent research activities on ticks and tick-borne diseases in Kazakhstan and Kyrgyzstan.

## Background

Biosurveillance research and environmental monitoring have been conducted in Central Asia since the Russian anti-plague (AP) network in the 1890s set up by Czar Nicholas II (1). This network was originally organized geographically, to conduct surveillance of local diseases, such as plague, and to prevent introduction of diseases with no natural foci, such as cholera (2). During the Soviet era, the AP network evolved into a highly structured organization that included over 100 facilities: observational, field, regional stations, and institutes, across the 11 republics that were administered by the Union of Soviet Socialist Republics’ Ministry of Health (3). After the Soviet Union collapsed, each country maintained its own AP system that varies by country (4). All have experienced challenges of limited funding and lack of training for their specialists, while competition for limited funds has decreased collaboration as well as development of programs fostering peer-reviewed quality research and related funding.

**Figure 1:**
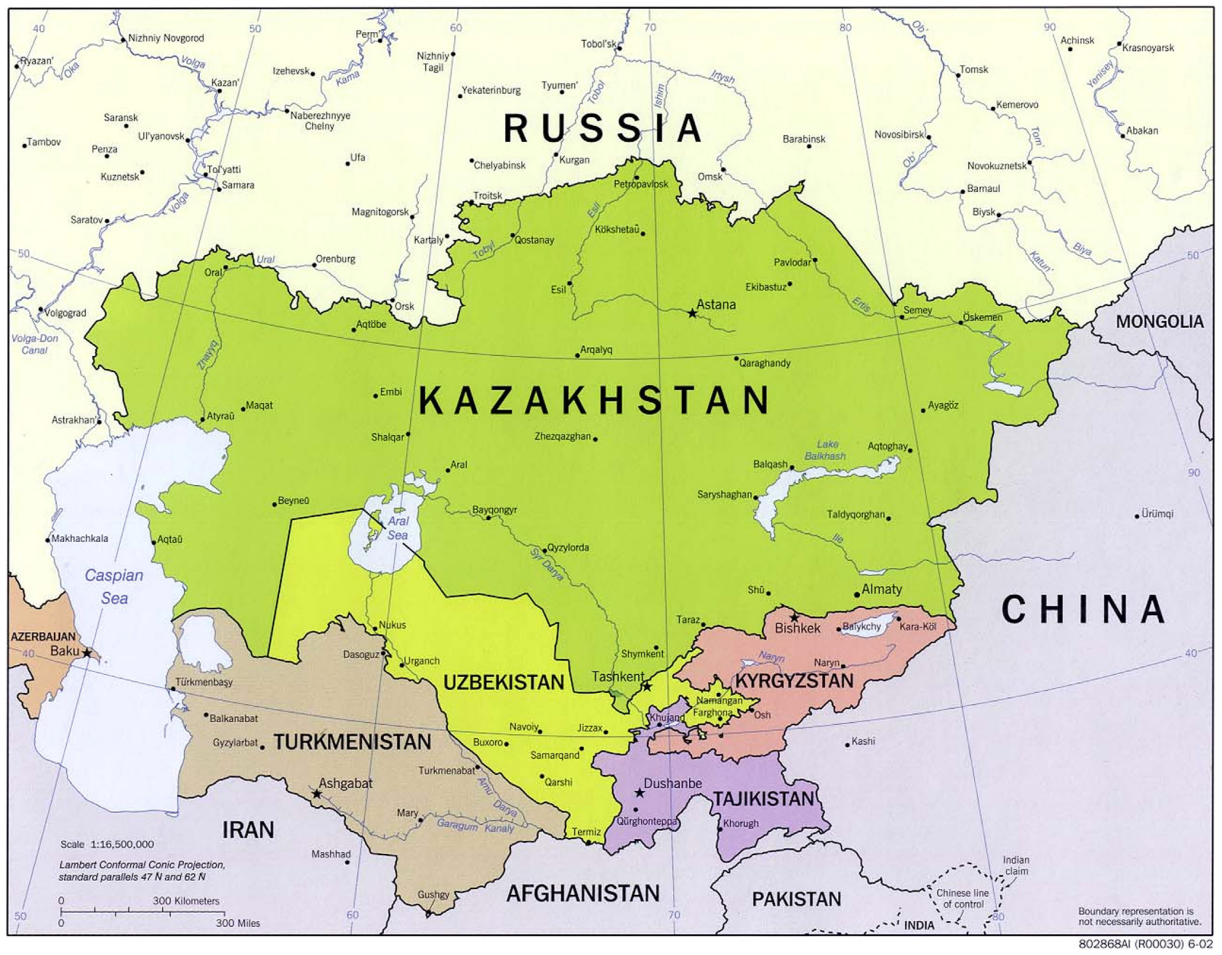
**Commonwealth of independent states – Central Asian States**. Reproduced with permission from (6).

Two countries that exemplify the current status of biosurveillance in Central Asia are Kazakhstan and Kyrgyzstan. Kazakhstan (2.7 million square kilometer; 18 million people) has national wealth in the forms of oil, natural gas, and mineral resources^1^ but still relies heavily on imported expertise and technology to further develop their resources (5). Kyrgyzstan (0.2 million square kilometer; 5.7 million people) has no oil or natural gas but has mineral resources (see text footnote 1), and also relies on imported expertise and technology (6) (Figure 1). In addition to commercial ventures with foreign petroleum companies, aid to both countries has come from the United States Department of Defense (US DoD) cooperative threat reduction (CTR) program, through the 1991 Soviet Nuclear Threat Reduction Initiative. The governments of both countries are cooperating on biological threat reduction efforts, with an emphasis on disease surveillance, biosafety, and biosecurity. As with other supporting nations, aid from the United States (US) first requires a formal government-to-government (umbrella) agreement, which establishes diplomatic intent and cooperation. Next, an implementing agreement is established with a specific US government agency. In Kazakhstan, an umbrella agreement was signed in December 1993 and the implementing agreement was signed in 1995. The cooperation includes a broad range of nuclear security and non-proliferation topics. Kyrgyzstan has also signed bilateral investment and trade agreements with the US. In Kyrgyzstan, multiple foreign agencies have been involved in projects utilizing the country’s AP system and run through the Republic Center of Quarantine and Especially Dangerous Infections (RCQEDI) (4). For example, International Science and Technology Center (ISTC), Civilian Research and Development Foundation (CRDF) Global, United States Department of State (US DoS), and the United Kingdom Ministry of Defense (UK MoD) have all set up collaborative biosurveillance projects with local scientists.

Historically, tick-borne diseases have been important public health issue in Central Asia, and biosurveillance has involved regular collection and archiving of tick samples. Collection locality information associated with these samples has already contributed to vector biogeography databases, such as VectorMap.^2^ Analyses of these frozen tick samples (e.g., molecular testing for evidence of infection by pathogens) offer numerous opportunities for additional research and collaboration, some examples of which are described below. In a recent paper, Han et al. (7) predicted the existence of a large reservoir of undiscovered zoonotic infections in this part of the world.

As examples of the accomplishments and challenges of biosurveillance research conducted in Central Asia we present below, as case studies, our work on ticks and tick-borne diseases conducted in Kazakhstan and Kyrgyzstan over the past decade. This work includes studies of tick-borne encephalitis (TBE) in both counties and of rickettsial diseases, Q fever, Crimean Congo hemorrhagic fever (CCHF), and hemorrhagic fever with renal syndrome (*Hantaviruses*) in Kazakhstan. For reasons of space limitation, we will not discuss the CCHF (8) or *Hantavirus* work in Kazakhstan (9) here.

## Tick-Borne Encephalitis

Tick-borne encephalitis is caused by a flavivirus (TBEV); there are three types: European, Siberian, and Far-Eastern. Of these, the Far-Eastern strain causes the most serious disease. Infection is spread to humans through tick bites and through ingestion of raw milk and milk products (10). Wild and domestic animals may be hosts for the virus (10). TBE is endemic in a wide range of European and Asian countries, and effective vaccines are available in many countries (11). Anecdotal information and papers in the local literature suggested that the virus and the disease may be more widespread than was understood, in particular, that the virus may range in more southerly locations in Asia (11, 12).

Based on this, we initiated a series of studies in Kyrgyzstan. We found TBEV in the taiga tick (*Ixodes persulcatus*) and in rodents including the Himalayan field mouse (*Apodemus pallipes*), a previously unknown TBEV host (13). Sequencing studies showed this Kyrgyz virus to be a close relative of a Siberian strain from Novosibirsk (13). We also identified the virus in a sample from a fatal case of TBEV involving a hiker who had visited an area where we found the virus in ticks (13). Thus, TBEV and TBE appear to occur much further south (42.6°N) and at much higher altitudes (~2,100 m) than previously believed (13).

Following this, we extended our TBE/TBEV research into Kazakhstan to identify and characterize TBEV there. This work, part of collaboration with Kazakhstani colleagues from three government agencies, has yielded several interesting findings over the past decade. Using modern molecular methods and an epidemiologically based approach, we tested human and animal samples from selected areas where TBE is known or suspected to occur and found strong evidence that TBEV does circulate in Kazakhstan Scientific colleagues from the Kazakh Scientific Center of Quarantine and Zoonotic Diseases (KSCQZD), Scientific Practical Center for Sanitary Epidemiological Expertise and Monitoring (SPCSEEM), and Uralsk Anti-Plague Station (UAPS) collaborated to provide samples and associated collection data, and assist with diagnostics. UAPS staff focused its work on tick samples from West Kazakhstan and Aktobe oblasts; KSCQZD staff collected ticks from Almaty, Kostanay, Kyzylorda, Mangystau, South Kazakhstan, and Zhambyl oblasts; and SPCSEEM staff obtained ticks from Atyrau, East Kazakhstan, Karaganda, North Kazakhstan, and Pavlodar oblasts. Collectively, tick samples included over 40,000 specimens collected from 13 of Kazakhstan’s 14 oblasts (i.e., provinces). Samples from captured rodents and stored human sera were shared by staff of UAPS and SPCSEEM, respectively. Our US-based staff provided technical guidance to Kazakh to facilitate confirmation of morphological tick identification by molecular methods [e.g., polymerase chain reaction (PCR) and pooling and testing of ticks for infection by TBEV, CCHFV, and *Rickettsia* spp. by enzyme-linked immunosorbent assay (ELISA) and quantitative real-time PCR (qPCR)].

As we hypothesized, TBEV is more widespread in Kazakhstan than was previously believed. Areas in the southern and eastern regions harbor infected ticks (14). Unfortunately, we were unable to get material from the western region, where we have strong suspicions that the virus also circulates. In the southern and eastern regions, data from 2005 to 2014 show, on average, 35 cases or 0.22 cases per 100,000 per year (15). This burden of disease apparently occurs in spite of the fact that up to 50,000 doses of vaccine are administered in endemic areas each year. Additionally, we found evidence of TBEV infection in ticks in the genera of *Dermacentor*, *Hyalomma*, and *Haemaphysalis* (14, 16, 17). The collective range of these ticks is much broader than that of *I. persulcatus*, suggesting that residents of a much broader area of Kazakhstan may be at risk for exposure to the virus (14). We are currently investigating the role of raw milk and cheese in the spread of TBE.

In summary, we have substantial new data on a serious tick-borne disease in Central Asia, of importance both to local and global public health authorities, as well as the US DoD. Acknowledging these successes, and the essential collaboration of the Kazakhstani and Kyrgyz authorities and our scientific colleagues, there are nevertheless issues that impede further development of our novel findings; these issues are discussed in the Section “Conclusion: Accomplishments and Challenges” below.

## Rickettsial Diseases

Historically, tick-borne rickettsial diseases in Kazakhstan were attributed solely to Siberian tick typhus. Siberian tick typhus is caused by *Rickettsia sibirica* subsp. *sibirica* that is transmitted by ixodid ticks (i.e., *Dermacentor* and *Haemaphysalis* spp.) (18). Between 2007 and 2012, 1,247 registered cases of Siberian tick typhus were recorded in Kazakhstan, with the highest prevalence (65%) occurring Kyzylorda and North Kazakhstan Oblasts (17). Whether these cases were truly due to Siberian tick typhus and/or other rickettsioses was not clear, because serological assays are cross-reactive for antibodies against the spotted fever group rickettsiae (SFGR) (19). Indeed, recent tick surveys have identified four additional SFGR species that may be responsible for rickettsial diseases in Kazakhstan (18, 20–22). These four agents, *Rickettsia conorii* subsp. *caspia*, *Rickettsia slovaca*, *Rickettsia raoultii*, and a *Rickettsia aeschlimannii*-like organism, were identified in collaborative studies between Russian and Kazakhstani scientists (18, 20–22).

Additional studies performed in Kazakhstan confirmed the presence of tick-borne rickettsiae among ticks collected through tick drags and small mammal trapping. During the spring of 2004, a study conducted by the UAPS in the southern tip of West Kazakhstan Oblast collected 33 *Rhipicephalus pumilio* ticks. Two ticks (6%) were positive for rickettsial DNA and data from subsequent sequencing analyses identified the agents as *R. conorii* subsp. *caspia*, the causative agent of Astrakhan spotted fever (17). Assessment of 330 ticks collected by tick drag in Karaganda Oblast in Central Kazakhstan by SPCSEEM determined that they were all *D. marginatus*. Of the 33 pools of these ticks tested for infection by rickettsial pathogens (10 ticks per pool), 2 from the Abai Rayon were positive (17).

Most recently (2012–2014), one large tick surveillance study supported by US Defense Threat Reduction Agency (DTRA) was conducted in Kazakhstan. One aspect of the investigation focused on detecting tick-borne rickettsiae and involved ticks collected in seven oblasts: Atyrau, East Kazakhstan, Zhambyl, Karaganda, Kyzylorda, Pavlodar, and West Kazakhstan involving SPCSEEM staff in Almaty (17) and UAPS staff in Uralsk (17, 23). The project explored both the identification of tick species and the detection of *Rickettsia* positive ticks by qPCR and multilocus sequencing typing (MLST); data analysis is ongoing.

## Q Fever

Q fever is caused by the Gram-negative bacterium, *Coxiella burnetii*, and is found all over the world (24). More than 40 species of ticks are known to be naturally infected with *C. burnetii*, and though tick-to-human transmission does occur it is only thought to be responsible for a small subset of infections (25). The most common route of human infection is through the inhalation of aerosolized particles, generally the result of the aerosolization of dried parturition materials from infected animals (26). Infected animals also shed *C. burnetii* in urine, feces, and milk, the last of which may cause infection when consumed without pasteurization (25). Q fever has been reported in Kazakhstan since the early 1950s (27). Currently, UAPS is conducting a study supported by DTRA assessing the presence of *C. burnetii* in unpasteurized milk in the West Kazakhstan Oblast utilizing a qPCR assay targeting IS1111 DNA (28).

## Conclusion: Accomplishments and Challenges

### Accomplishments

During the last decade in Kazakhstan and Kyrgyzstan, we have carried out extensive training and research work in collaboration with our local colleagues. These efforts have led to enhanced biosafety and biosecurity practices, successful implementation of field and laboratory techniques, and awareness and compliance with regulatory standards. Modern rodent capture methods were also introduced, which included training on the use of live capture traps and safe rodent handling. As a result, we have been able to develop technical presentations in a number of research areas, many of which are cited in the reference list.

Our collaborative studies have also generated extensive collections of field samples and associated analytical datasets. For example, we now have over 40,000 ectoparasites from tick drags and vertebrate hosts, many of which have been identified both by morphological and molecular means. Data, including tick species, location (GPS coordinates), and date of collection were recorded and shared via public databases.

In the laboratory, enhanced sample processing and use of molecular diagnostics were emphasized. Safe and proper procedures for bead-beating tick samples were employed, and nucleic acid extraction (DNA and RNA) was performed on those samples before qPCR determination of bacterial and viral identity. We also demonstrated and implemented methods that comply with Federal Wide Registration (FWA) standards for testing of stored human sera.

Finally, between 2011 and 2014, our work in central Asia produced 20 scientific papers presented at 11 professional conferences. These presentations were developed in collaboration with our in-country colleagues. In addition, one peer-reviewed manuscript based on this work has been published to date (8).

Overall, these considerable accomplishments have resulted in a better trained and aware scientific workforce in Kazakhstan and Kyrgyzstan. Our central Asian colleagues have been exposed to modern scientific methodology and perspectives and have been able to present their work in several major international forums. This has led to significant findings in public health in this part of Central Asia, which will increase awareness of important infections locally and globally. Finally, this work will provide a solid basis for future planning for infectious disease surveillance and research in Central Asia.

### Challenges

In the course of all the successful work described above, we encountered a number of challenges that are being addressed to facilitate continued progress in Kazakhstan and Kyrgyzstan. From the literature, one can see that only a few surveyed locations in this region are reported in peer-reviewed international journals. Even from the last large study that surveyed many oblasts, the number of rayons (i.e., county or district) studied within each oblast was limited, and thus the overall investigation is constrained. An obvious solution would be to continue and expand biosurveillance studies, varying the sites over time, but is time consuming and expensive.

Another issue is the limited submission of research results to peer-review, high impact international journals, especially English language journals, by local scientists. Scientists around the world are developing the scientific credentials through successful submission of their results to peer-review journals. This submission process needs to be encouraged for scientists in Central Asia, especially in regards to writing detailed results and statistics required for publication in peer-reviewed, international journals. In addition, the development of this writing skill and the publication of notable results will enhance the success at developing and submitting proposals for local and international grant funding.

A further crucial area for improvement is the development of technical skills for assessing and developing assays and methods, as well as data analysis. Lack of standard operating procedures in Russian, Kazakh, and Kyrgyz languages, and trained technical personnel are other problems. Current reliance on commercial test assays is costly and unsustainable. Possible solutions to these problems include developing reagent/equipment procurement streams via known in-country and/or regional commercial entities and developing in-house assays that can be compared with currently accepted commercial assays. Increasing molecular diagnostics capabilities at institutions, including government, university, and commercial entities, would also be useful, particularly if these could be shared with among institutions. Limited submission of samples for analysis and verification to outside institutions lessens future progress. This issue can only be solved by allowing scientists to bring or send test sample material to foreign institution; this will not be simple to resolve and will require substantial political will on the part of the local authorities.

Finally, there are many communication barriers to overcome. This would be helped by including each level of work group (including technicians, scientists, managers, and directors) in discussions of research activity, as well as encouraging clear communication within organizations to improve research and planning. In our work, we found that direct communication, especially face-to-face meetings and conference calls, was most effective and decreased the need for written correspondence that required language translation.

Overall, the accomplishments in the biosurveillance studies conducted in Kazakhstan and Kyrgyzstan presented here demonstrates that significant work can be achieved despite existing challenges. With time and continued collaborative efforts, we believe that these challenges will overcome, leading to even more progress in the future. Current interest in global health security activities coupled with the historic zoonotic diseases in this part of the world provides ample opportunities for further studies.

## Conflict of Interest Statement

The authors declare that the research was conducted in the absence of any commercial or financial relationships that could be construed as a potential conflict of interest. The Reviewer Brad S. Schneider and handling Editor Nathan Wolfe declared their shared affiliation, and the handling Editor states that the process nevertheless met the standards of a fair and objective review.

## Footnotes

^1^https://www.cia.gov/library/publications/the-world-factbook/geos/kg.html

^2^http://www.vectormap.org/dataportal.htm
